# Supplementary material for: Salmonella in Captive Reptiles and Their Environment—Can We Tame the Dragon?
Source: Microorganisms. 2021 May 8;9(5):1012. doi: 10.3390/microorganisms9051012 (PMC8151916; doi:10.3390/microorganisms9051012)
Supplement: Supplementary file 1 [file microorganisms-09-01012-s001.zip › microorganisms-1184542-supplementary.pdf]

Table S1. *Salmonella* serovars (n =151) isolated from different media from single samples: from MSRV, both from MSRV and RSA and only from RSA. Serovars obtained exclusively from RSA (n = 25) were bolded.

Salmonella serovar	MSRV	MSRV and RSA	RSA	Total
S. Newport	2	2	8	12
S. II 58:a:z6	1	1	8	10
S. II 30:l,z28:z6	22	13	7	42
S. Fluntern	14	15	5	34
***S.* II 50:b:z6**	**0**	**0**	**4**	**4**
S. Tennessee	10	13	3	26
S. IIIb 53:z10:z35	1	4	3	8
S. Hadar	2	1	3	6
S. Monschaui	2	0	3	5
S. IIIb 14:z10:z	1	0	3	4
S. Muenchen	11	6	2	19
S. Pomona	7	2	2	11
S. Ago	5	1	2	8
S. II 16:m,t:-	3	1	2	6
S. IIIb 47:k:z35	3	0	2	5
S. Paratyphi B v.Java	3	0	2	5
S. Aqua	1	1	2	4
S. IIIb 57:k:enxz15	0	1	2	3
***S.* IIIb 59:k:z**	**0**	**0**	**2**	**2**
***S.* IIIb 59:z52:z53**	**0**	**0**	**2**	**2**
S. Agona	16	8	1	25
S. Oranienburg	18	4	1	23
S. Kentucky	11	4	1	16
S. II 1,40:g,m,t:-	10	3	1	14
S. Infantis	4	8	1	13
S. IIIa 41:z4,z23:-	5	3	1	9
S. Adelaide	4	1	1	6
S. Typhimurium	2	2	1	5
S. Florida	3	0	1	4
S. IV 44:z4,z23:-	1	2	1	4
S. Apapa	2	0	1	3
S. Gatuni	1	1	1	3
S. IIIb 48:i:z	2	0	1	3
S. IIIb 61:z52:z53	1	1	1	3
S. IV 45:g,z51:-	2	0	1	3
S. I 4:eh:-	0	2	1	3
S. Blijdorp	1	0	1	2
S. Bolombo	1	0	1	2
S. Inverness	1	0	1	2
S. Montevideo	0	1	1	2
S. Oslo	0	1	1	2
S. II 58:l,z13,z28:z6	0	1	1	2
S. IIIb 50:z52:z53	1	0	1	2
S. IV 43:z4,z23:-	1	0	1	2
***S.* Bispebjerg**	**0**	**0**	**1**	**1**

***S.* Kisarawe**	**0**	**0**	**1**	**1**
***S.* Lisboa**	**0**	**0**	**1**	**1**
***S.* Overschie**	**0**	**0**	**1**	**1**
***S.* Reading**	**0**	**0**	**1**	**1**
***S.* IIIb 61:i:z**	**0**	**0**	**1**	**1**
***S.* II 58:l,z13,z28:-**	**0**	**0**	**1**	**1**
***S.* sp. (rough) IIIb**	**0**	**0**	**1**	**1**
***S.* IIIb 48:r:z**	**0**	**0**	**1**	**1**
***S.* II 21:z10:-**	**0**	**0**	**1**	**1**
***S.* IIIb 47:z10:z35**	**0**	**0**	**1**	**1**
***S.* IIIb 18:l,v:z**	**0**	**0**	**1**	**1**
***S.* IIIb 50:r:z**	**0**	**0**	**1**	**1**
***S.* IIIb 35:i:z35**	**0**	**0**	**1**	**1**
***S.* IIIb 38:k:1,5,7**	**0**	**0**	**1**	**1**
***S.* IIIb 58:r:z53**	**0**	**0**	**1**	**1**
***S.* IIIb 11:l,v:z**	**0**	**0**	**1**	**1**
***S.* II 17:g,t:-**	**0**	**0**	**1**	**1**
***S.* IV 41:z4,z23:-**	**0**	**0**	**1**	**1**
***S.* IV 50:g,z51:-**	**0**	**0**	**1**	**1**
***S.* I 6,8:-:-**	**0**	**0**	**1**	**1**
***S.* I 4,5:b:-**	**0**	**0**	**1**	**1**
S. Poano	7	1	0	8
S. Carrau	4	1	0	5
S. Cubana	1	4	0	5
S. Enteritidis	4	0	0	4
S. Kintambo	2	2	0	4
S. Miami	4	0	0	4
S. Nima	4	0	0	4
S. Benin	2	1	0	3
S. Ekpoui	3	0	0	3
S. Fresno	3	0	0	3
S. Jangwani	3	0	0	3
S. Teddington	2	1	0	3
S. IIIa 13,23:z4,z23,z32:-	1	2	0	3
S. IV 42:z36:-	1	2	0	3
S. I 45:b:-	3	0	0	3
S. Cotham	1	1	0	2
S. Manhattan	2	0	0	2
S. Muenster	2	0	0	2
S. Mundonobo	1	1	0	2
S. Poona	2	0	0	2
S. Rosslyn	2	0	0	2
S. Urbana	1	1	0	2
S. II 21:g,t:-	1	1	0	2
S. II 21:z10:z6	1	1	0	2
S. IIIb 50:k:z		2	0	2
S. IIIb 38:r:z	2	0	0	2
S. IIIb 47:r:z53	1	1	0	2
S. IV 48:g,z51:-	2	0	0	2
S. IV 16:z4,z32:-	2	0	0	2

S. IV 38:z4,z23:-	2	0	0	2
S. Abony	0	1	0	1
S. Anatum	0	1	0	1
S. Baildon	0	1	0	1
S. Bareilly	1	0	0	1
S. Blukwa	1	0	0	1
S. Braenderup	1	0	0	1
S. Chicago	0	1	0	1
S. Choleraesuis var. Decatur	1	0	0	1
S. Durban	1	0	0	1
S. Gaminara	1	0	0	1
S. Glostrup	1	0	0	1
S. Hofit	1	0	0	1
S. Itami	1	0	0	1
S. Jodhpur	1	0	0	1
S. Johannesburg	1	0	0	1
S. Labadi	1	0	0	1
S. Lattenkamp	0	1	0	1
S. Lome	1	0	0	1
S. Madelia	1	0	0	1
S. Mountpleasant	1	0	0	1
S. Orlando	1	0	0	1
S. Patience	1	0	0	1
S. Sandiego	1	0	0	1
S. Senftenberg	1	0	0	1
S. Tanzania	1	0	0	1
S. Telelkebir	1	0	0	1
S. Toucra	0	1	0	1
S. Treforest	0	1	0	1
S. Uzaramo	0	1	0	1
S. IIIb 35:k:z53	1	0	0	1
S. IIIb 57:lv:z35	1	0	0	1
S. IIIb 6,14:z10:z	0	1	0	1
S. II 9,46:z10:-	1	0	0	1
S. IIIa 44:z4,z32:-	0	1	0	1
S. IIIa 42:z4,z24:-	1	0	0	1
S. II 9:z29:1,5	1	0	0	1
S. IIIb 48:k:z53	0	1	0	1
S. IIIb 50:k:z:z57	1	0	0	1
S. IIIa 13,23:z4,z32:-	1	0	0	1
S. IIIb 48:z52:z	0	1	0	1
S. IIIa 48:g,z51:-	1	0	0	1
S. IIIa 44:z4,z24:-	1	0	0	1
S. IIIa 44:z4,z23:-	0	1	0	1
S. IIIb 50:i:1,5,7	1	0	0	1
S. IIIa 44:z4,z23,z32:-	0	1	0	1
S. IIIa 48:z4,z24:-	1	0	0	1
S. II 43:g,m,t:-	0	1	0	1
S. IIIa 40:z4,z23,z32:-	1	0	0	1
S. II 47:a:1,5	1	0	0	1

S. IIIb 50-:-	1	0	0	1
S. IV 44:z4,z24:-	1	0	0	1
S. V 48:z65:-	1	0	0	1
S. sp. I	1	0	0	1
S. sp. (rough) I	1	0	0	1
S. I 35:-:-	1	0	0	1
Total	281	140	113	534
